# Supplementary material for: Insights on Gut and Skin Wound Microbiome in Stranded Indo-Pacific Finless Porpoise (Neophocaena phocaenoides)
Source: Microorganisms. 2022 Jun 27;10(7):1295. doi: 10.3390/microorganisms10071295 (PMC9318903; doi:10.3390/microorganisms10071295)
Supplement: Supplementary file 1 [file microorganisms-10-01295-s001.zip › microorganisms-1739287-supplementary.pdf]

Novel insights on gut and skin wound microbiome in Indo-Pacific finless porpoise

Chengzhang Li[1,2], Huiying Xie[1,2#], Yajing Sun[1,2], Ying Zeng[1,2], Ziyao Tian[1,2], Xiaohan Chen[1,2], Edmond Sanganyado[1,2,3], Jianqing Lin[1,2], Liangliang Yang[1,2], Ping Li[1,2], Bo Liang[1,2*], Wenhua Liu[1,2*]

[1] *Guangdong Provincial Key Laboratory of Marine Biotechnology, Institute of Marine Science, Shantou University, Shantou, Guangdong, 515063, China;*

[2] *Southern Marine Science and Engineering Guangdong Laboratory, Guangzhou 511458, China;*

[3] *Department of Applied Sciences, Northumbria University, Newcastle upon Tyne NE1 8ST, UK*

# Co-first author

*Corresponding author: whliu@stu.edu.cn (W. Liu), liangbo@stu.edu.cn (B. Liang)

Tables

Supplementary Table S1, health status assessment by post-mortem examinations.

Sex	Weight (kg)	Body Length (cm)	Age	Blubber thickness (mm) *	
				sternal	caudoventral to the dorsal fin
Female	45	115	subadult	21	32

* Reference values for blubber thickness: 20–50 mm (good health status), 15–30 mm (moderate health status), 5–20 mm (poor health status) [1].

Figures Legends

Supplementary Figure S1, Composition and abundance of microbiome from GI tract and skin wound. (A) The rarefaction curve during the sequencing. (B) Venn diagram of OTUs.

Supplementary Figure S2, relative abundance of class Gammaproteobacteria, order Vibrionales, family Vibrionaceae, and genera Photobacterium of GI tract samples.

Supplementary Figure S3, relative abundance of genera Photobacterium (A) and Actinobacillus (B) of GI tract samples.

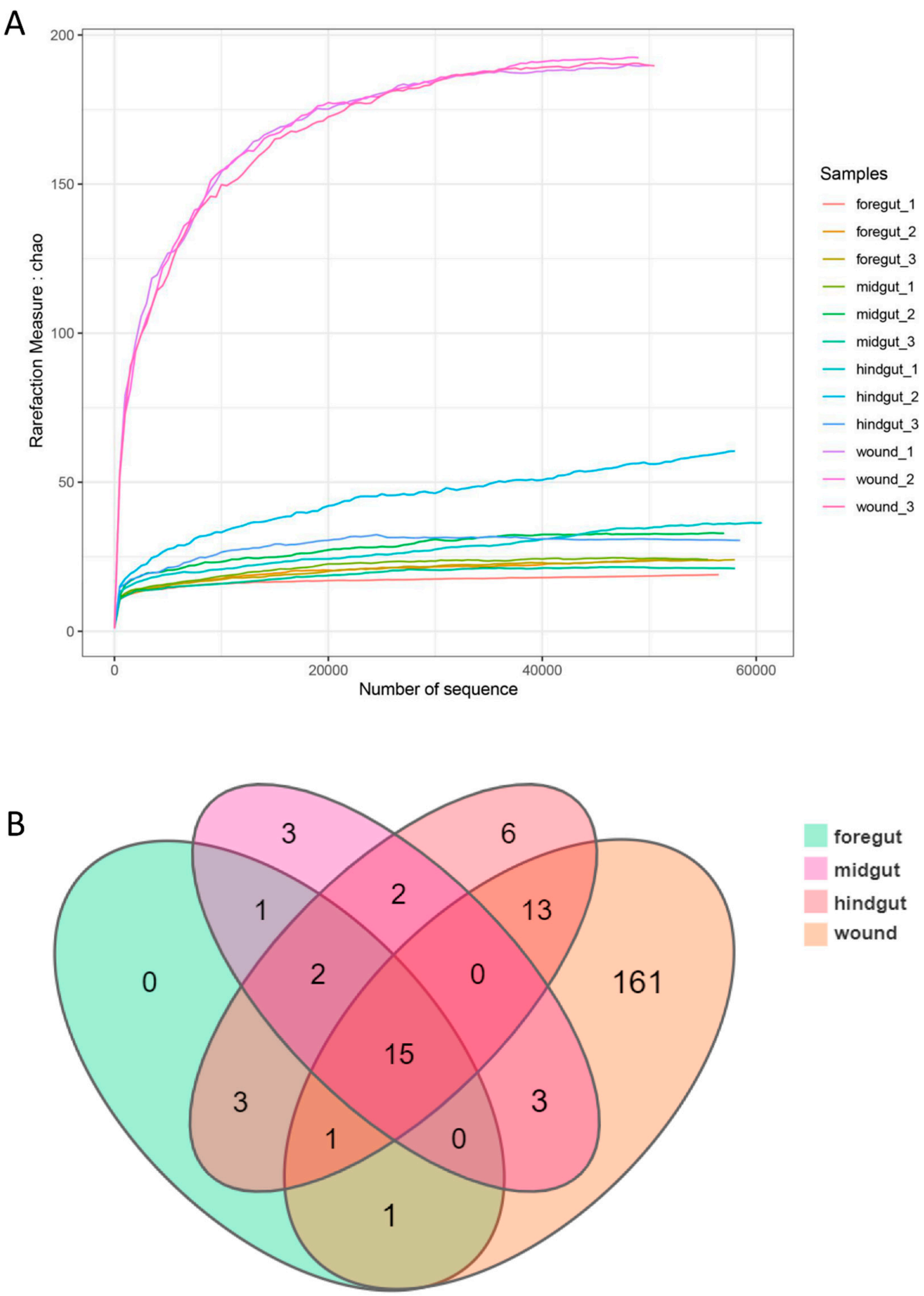

Supplementary Figure S1,

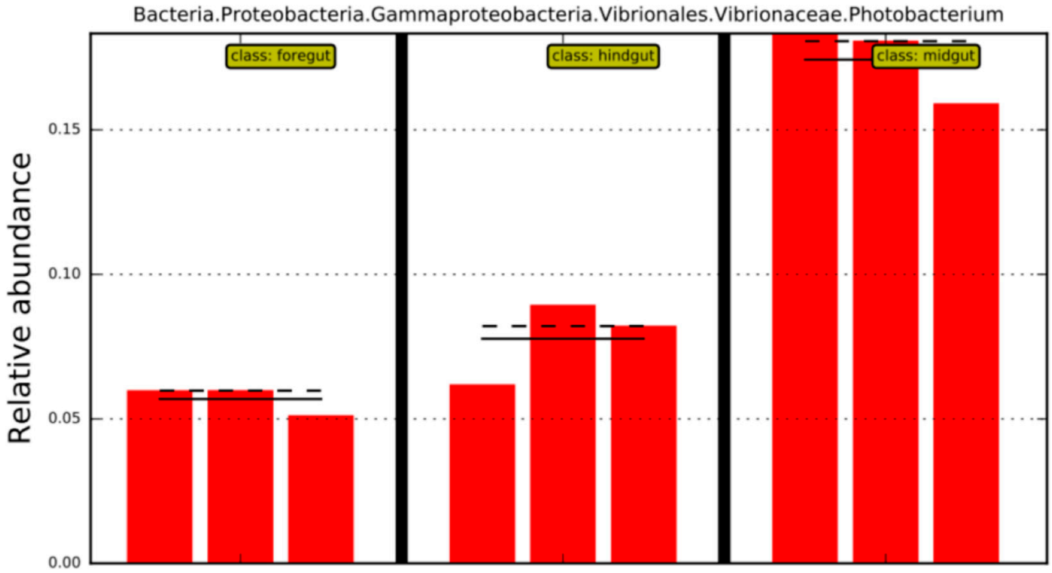

Supplementary Figure S2

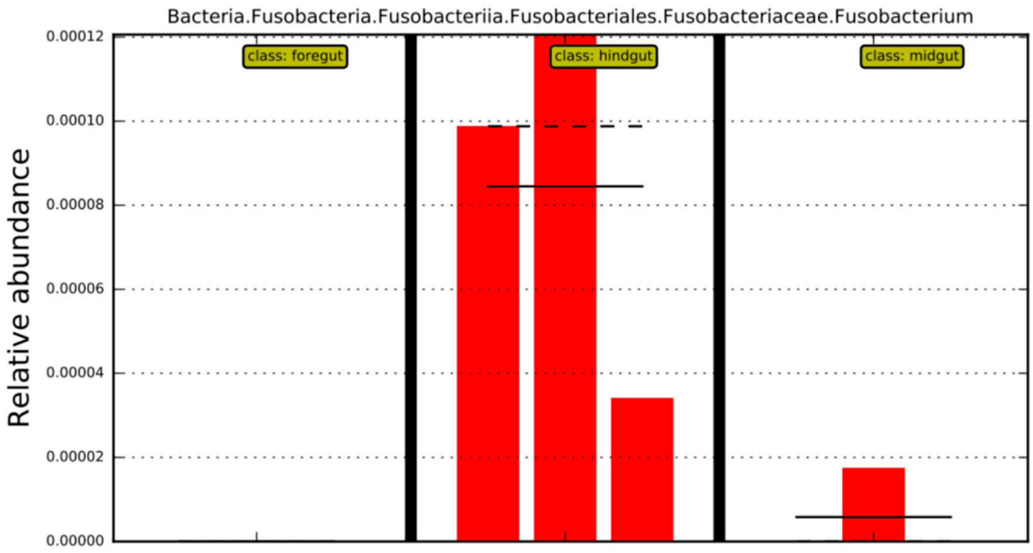

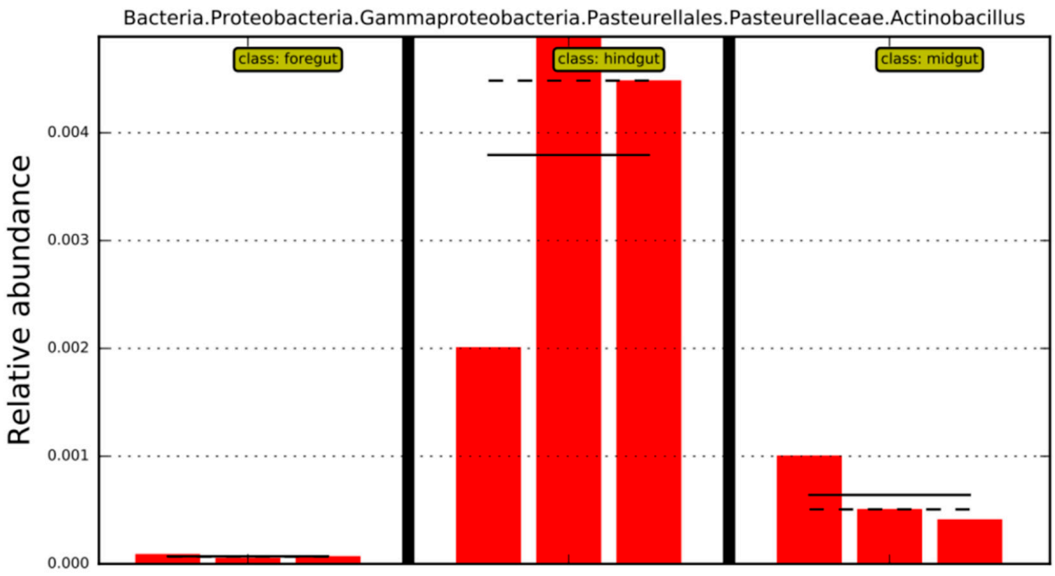

Supplementary Figure S3

Reference

1. Siebert U, Wünschmann A, Weiss R, Frank H, Benke H, Frese K (2001) Post-mortem Findings in Harbour Porpoises (Phocoena phocoena) from the German North and Baltic Seas. Journal of Comparative Pathology 124 (2):102-114. doi:https://doi.org/10.1053/jcpa.2000.0436.
